# Up-regulation of multiple proteins and biological processes during maxillary expansion in rats

**DOI:** 10.1186/1471-2474-9-37

**Published:** 2008-03-19

**Authors:** Junqing Ma, Yunxia Wu, Weibing Zhang, Roger J Smales, You Huang, Yongchu Pan, Lin Wang

**Affiliations:** 1 Institute of Stomatology, School of Stomatology, Nanjing Medical University, PR China; 2 School of Dentistry, Faculty of Health Sciences, The University of Adelaide, Adelaide 5005, Australia

## Abstract

**Background:**

Maxillary expansion (ME) is a common practice in orthodontics that aims to increase the constricted maxillary arch width. Relapse often occurs, however, and better treatment strategies are needed. In order to develop a more effective method, this study was designed to further examine the process of tissue remodeling during ME, to identify the changes in expression of several proteins of interest, and to clarify the molecular mechanism responsible for tissue remodeling.

**Methods:**

Male Wistar rats were randomly divided into control and ME groups. The rats were euthanized at various intervals over 11 days, and the dissected palates were prepared for histological examination. The structure of the midpalatal sutures changed little during the first three days. Proteins from samples in the ground midpalatal tissues obtained on the third day were subjected to two-dimensional polyacrylamide gel electrophoresis (2-DE) and matrix assisted laser desorption/ionization-time of flight mass spectrometry (MALDI-TOF MS) analysis. Validation of protein expression was performed by Western blot analyses.

**Results:**

From day 5, chondrocytes in the inner layer of suture cartilage and osteoblasts at the end of the suture cartilage began to proliferate, and the skeletal matrix increased later adjacent to the cartilage in the ME group. Comparative proteomic analysis showed increases in 22 protein spots present in the ME group. The changes in three proteins closely related to osteogenesis (parathyroid hormone, osteoprotegerin and vimentin) were confirmed by Western blotting.

**Conclusion:**

Many proteins are over-expressed during ME, and they may play an important role in the remodeling process.

## Background

Maxillary expansion (ME), or palatal expansion, is a common practice in orthodontics that aims to increase constricted maxillary arch width. The procedure is often performed to treat posterior crossbites, and is sometimes used in instances of arch crowding, Angle Class I malocclusions with a V-shaped maxillary arch, and Angle Class III malocclusions with a maxillary deficiency. However, even after long-term retention is applied to prevent a relapse, there is generally a reduction of the expanded maxillary arch width to some extent [1].

Several studies in rats and other animals have been undertaken to explore the mechanism of tissue remodeling in order to improve the efficacy of ME. The expressions of TGF-β1 [2], integrin and FAK [3] in ME have been elucidated, and several adjunctive ME therapies have been proposed including administration of TGF-β1 [2], low-power laser irradiation [4], bisphosphonate [5], BMP-7 and Nell-1 [6].

ME is a special form of sutural distraction osteogenesis in which a mechanical force is transformed into a biological stimulus, which initiates tissue remodeling and new bone formation in the midpalatal suture. The complicated biological process may involve chondrocyte hypertrophy, angiogenesis, absorption of original tissues in the suture, and the formation of skeletal matrix. Many proteins may contribute to the final results. Previous studies have been limited to investigating one or a few proteins, but the complex biological mechanism of ME necessitates the use of a global proteomic analysis to improve the understanding of the process in greater detail.

Proteomics is the study of all proteins expressed by genomes, and provides a global analysis of complex protein mixtures. Proteomic methodologies for differentially expressed profiles of tissue proteins from the midpalatal sutures of a ME group and a control group may provide clues about the biological functions of these proteins during ME. The present study was designed to obtain a further understanding, via differential proteomics evaluations, of tissue remodeling during ME and to determine whether any proteins are differentially expressed, and whether these proteins are related to the observed tissue remodeling.

## Methods

### Experimental animals

Six-week-old male Wistar rats were procured from the Shanghai SLAC Laboratory Animal Co. Ltd and bred in the Nanjing Medical University Animal Center (NJMUAC). The pre-operative and post-operative care of these animals was overseen by NJMUAC veterinarians to ensure proper and humane treatment. The rats were all fed commercial pellet food with water ad libitum, and were housed in cages under controlled conditions at 25°C on a 12h:12h light/dark cycle (light cycle starting at 7:00 a.m.). The health status of each rat was evaluated by daily body weight monitoring. Approval for the study was obtained from the Animal Ethics Committee of Nanjing Medical University.

### ME procedure

The rats were randomly divided (RandA1.0 Software, Planta Medical Technology and Development Co. Ltd, Beijing, PR China) into an experimental ME group and a control group (52/group). After being anesthetized by an intraperitoneal injection of sodium pentobarbital at 50 mg/kg body weight, the 52 rats in the experimental group received the ME operation. Briefly, a 1.5 mm thick circular stainless steel expander ring was inserted between the maxillary incisors and held by a 0.2 mm diameter round wire on the first day of the experiment using the method reported in previous studies [2,4]. From each group, Twenty-eight rats were randomly selected for subsequent histological examination and 24 for two-dimensional polyacrylamide gel electrophoresis (2-DE).

### Histological examination

Four rats from each group were euthanized by overdoses of sodium pentobarbital at various times: before operation (0 d), or 1 d, 3 d, 5 d, 7 d, 9 d and 11 d after the operation. The midpalatal suture tissues were dissected and the specimens obtained were decalcified, paraffin-embedded, sectioned and stained with haematoxylin-eosin and Masson's trichrome before examination under a microscope.

### Immunohistochemical analysis

The paraffin sections were dewaxed and pre-treated with 3% hydrogen peroxide. Expression of proliferation cell nuclear antigen (PCNA) and Collagen I were detected using the Streptavidin-Biotin-Peroxidase Complex (SABC) immunohistochemical method (PCNA SABC kit, Boster Biotechnology, Wuhan, PR China). The goat anti-rat Collagen I monoclonal antibody was obtained from Santa Cruz Biotechnology (Santa Cruz, CA, USA).

### Two-dimensional polyacrylamide gel electrophoresis (2-DE)

**Figure 1:**
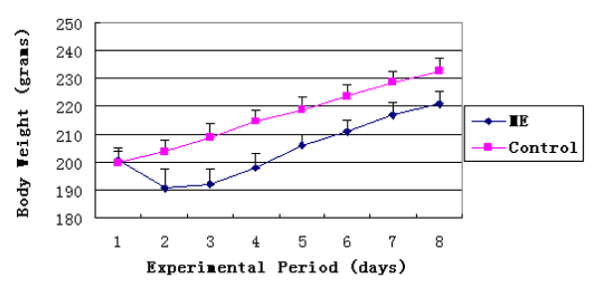
Changes in rat body weight in the control and ME groups. ME, Maxillary Expansion group. Control, untreated animals.

The health status of the rats in the ME group was evaluated by monitoring daily body weights that were recovered from the third day (Fig 1). Since significant histological changes in the sutures were observed starting on the fifth day after operation, we chose to perform the proteomic analysis of the midpalatal sutures 3 d after the operation (24 rats/group) to evaluate which proteins might be responsible for the observed structural changes. The rats were euthanized as described above and the midpalatal suture tissues were dissected and stored on ice. The fresh tissue specimens were rinsed in ice-cold 0.01 M PBS buffer solution, and then ground into powder in liquid nitrogen. Proteins were extracted by suspending the tissue powder in a sample buffer (7 M urea, 2 M Thiourea, 4% CHAPS, 100 mM DTT, 40 mM Tris, 1 mM PMSF) at room temperature and vortexing for 2 min. They were then stored at 4°C overnight. The supernatant was concentrated by using a ReadyPrep 2-D Cleanup Kit (Bio-Rad, Hercules, USA), removing impurities such as salts. The sediments were re-dissolved in a sample buffer (7 M urea, 2 M Thiourea, 4% CHAPS, 100 mM DTT, 40 mM Tris, 1 mM PMSF, 0.2% Carrier Ampholyte (pH 3–10), trace of bromophenol blue) and incubated at room temperature for 2 h. Protein concentrations were determined by the Bradford protein assay (Bio-Rad).

First-dimension isoelectric focusing (IEF) was performed with precast 17-cm immobilized pH gradient (IPG) strips (17 cm ReadyStrip IPG Strip 3–10 NL, Bio-Rad). Three protein samples from each group were loaded, respectively, onto the PROTEAN IEF Focusing Tray (Bio-Rad) containing 300 μl of a mixture of the rehydration buffer (7 M urea, 2 M Thiourea, 4% CHAPS, 100 mM DTT, 0.2% Carrier Ampholyte (pH 3–10), trace of bromophenol blue) and the sample buffer containing 80 μg protein. After in-gel rehydration at 20°C for 12 h, proteins were focused at 250 V at linear ramp for 0.5 h, 1,000 V at rapid ramp for 1 h, 10,000 V at linear ramp for 5 h, and 10,000 V at rapid ramp for 6 h. All of the above procedures were performed at 20°C. Immediately after IEF, the IPG strips were equilibrated in the first equilibration buffer (6 M urea, 2% SDS, 375 mM Tris-HCl (pH 8.8), 20% glycerol, 2% (w/v) DTT) for 15 min, and the second equilibration buffer (6 M urea, 2% SDS, 375 mM Tris-HCl (pH 8.8), 20% glycerol, 2.5% (w/v) iodoacetamide) for another 15 min.

After the IPG Strips were rinsed with the 1 × Tris-glycine-SDS running buffer, they were placed on 12% SDS-PAGE gels and sealed with 1% LowMelt agarose. Second-dimension SDS-PAGE was carried out in a PROTEAN Plus Dodeca Cell (Bio-Rad) at 100 V for 0.5 h, followed by electrophoresis at 200 V, until the bromophenol blue front reached the bottom of the gels. The protein spots on the gels were fixed and visualized by silver staining and scanned at 600 dpi. Analysis of the gel images for differentially-expressed proteins was carried out using PDQuest v7.3 software (Bio-Rad, Hercules, USA).

### Mass spectrometry analysis and protein identification

The differentially-expressed protein spots were manually excised into 1-mm^2 ^pieces from the silver-stained gels. The gel pieces containing the protein spots were destained by rinsing three times with 25 mM ammonium bicarbonate in 50% (v/v) acetonitrile for 45 min each. They were dried at 50°C for 20 min, then subjected to in-gel digestion with 30 μl of digestion solution (12.5 μg/ml trypsin in 25 mM ammonium bicarbonate) at 37°C for 16 h.

The peptides were concentrated by vigorous shaking at room temperature, using a modification of the method developed by Shevchenko [7]. Briefly, 30 μl of 25 mM ammonium bicarbonate was added to the digestion solution mixture for 15 min, followed by the addition of 30 μl of 100% (v/v) acetonitrile for another 15 min, after which the supernatant was collected. The extraction procedure was repeated two more times with 5% (v/v) formic acid in 100% (v/v) acetonitrile. The supernatants were pooled and dried in a Speed-Vac (Thermo Savant, Holbrook, USA). The extracted peptides were then re-dissolved in 20 μl of 2% (v/v) formic acid. The peptide fingerprint spectra of proteins were obtained by matrix-assisted laser desorption/ionization-time of flight mass spectrometry (MALDI-TOF MS, Bruker Inc, Billerica, USA) and were identified in the rattus protein database using the MASCOT search engine [8]. The Swiss-Prot, Trembl and NCBI databases were used to obtain further information about the proteins identified.

### Validation by Western blot

Twenty-two proteins were detected whose expression was substantially increased in the ME group by 2-DE and MALDI-TOF MS. Among them, three proteins (parathyroid hormone (PTH), osteoprotegerin (OPG), and vimentin) may have an important role in the development of osteogenesis and therefore were chosen for validation by Western blot analysis. Western blotting was performed as described previously [9]. The initial sample for immunoblotting was prepared in the same way as for 2-DE. β-actin was used as a loading control. The separated proteins were incubated overnight with primary anti-PTH, anti-OPG and anti-vimentin antibodies (Santa Cruz Biotechnology, CA, USA). The blots were then incubated with peroxidase-conjugated secondary antibody (1:2,000). Protein bands were visualized using an enhanced chemiluminescence system (Supersignal West Pico Trial Kit, Pierce, Rockford, USA) and the density of each band was quantified with a Fluor-S MultiImager (Bio-Rad). The experiment was performed with samples from six rats in each group and the mean expression level for each protein was calculated accordingly.

## Results

### Effect of ME on body weight

Compared with the control group, the body weights of the rats in the ME group decreased during the first two days after operation and started recovering on the third day (Figure 1). The average weights of the rats in the two groups were significantly different from the second day to the end of the experiment (P < 0.05). However, there was only an approximately 5% difference in the mean weights of the two groups at the end of the study period. This small difference was caused by initial weight loss in the ME group during the first two days as a result of the experimental procedure. There were no statistically significant differences between the two groups in daily weight gain from the second day (P > 0.05).

### Histological alterations in the midpalatal sutures caused by ME

**Figure 2:**
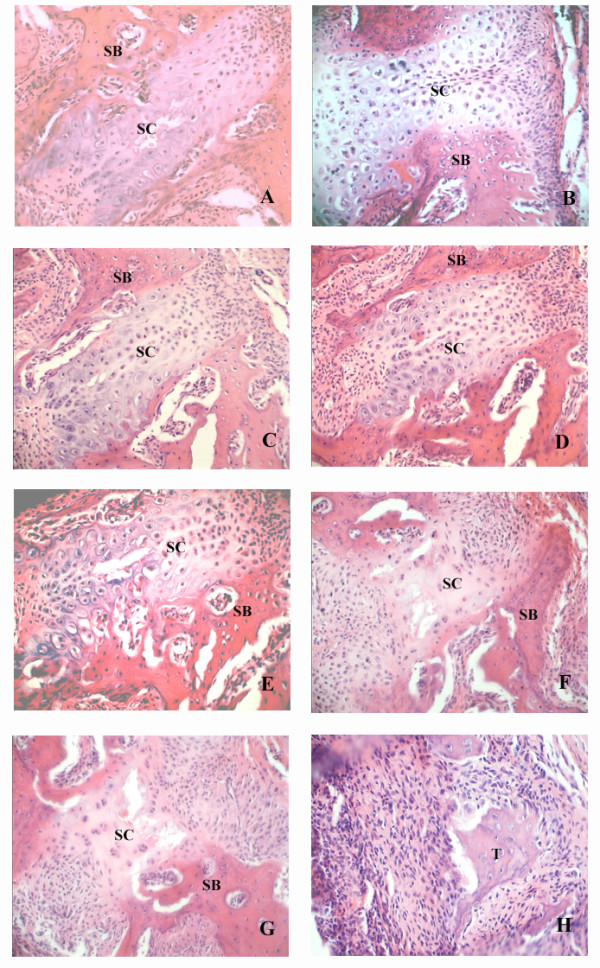
Photomicrographs of midpalatal sutures during ME. A: 0 d control group, B: 9 d control group, C: 0 d ME group, D: 1 d ME group, E: 3 d ME group, F: 5 d ME group, G: 7 d ME group, and H: 9 d ME group. SC: suture cartilage. SB: suture bone. There were no significant differences between the two groups in the tissue structure of the midpalatal sutures before day 5, but from day 5 until the end of the study, there was significant tissue remodeling observed in the sutures of the ME group that was not observed for the control animals. (Haematoxylin-eosin, original magnification ×40). The tissue marked " T " was further validation as bone or cartilage by determination of Collagen I localization (Figure 5).

**Figure 3:**
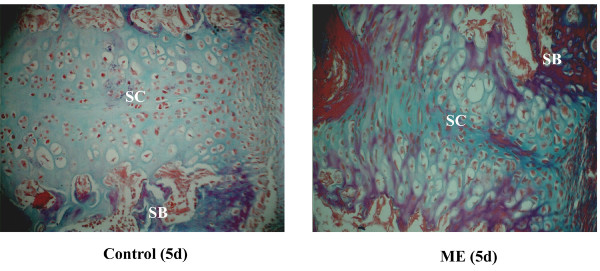
Photomicrographs of the midpalatal sutures on day 5. SC: suture cartilage. SB: suture bone. Much more skeletal matrix (purple) is present adjacent to the suture cartilage in the ME group than in the control group. (Masson's trichrome, original magnification ×40).

In the control group, histological observations of the sutures showed no remarkable changes throughout the study (Figs 2A, B), and were similar to the sutures of rats in the ME group on day 0 (Figure 2C). In the ME group, during the first three days after operation, there were no significant changes in the the cellular composition and structure of the midpalatal sutures (Figs 2C, D, E). But, starting on day 5 there was significant tissue remodeling observed in the sutures of the ME group, including absorption of suture cartilage and invasion of new tissue (Figs 2F, G, H). Masson's trichrome staining showed that more newly-formed skeletal matrix was present between the cartilage and bone in the ME group than in the control group (Figure 3), indicating a critical role of endochondral ossification in the ME process.

### PCNA and Collagen I expression

**Figure 4:**
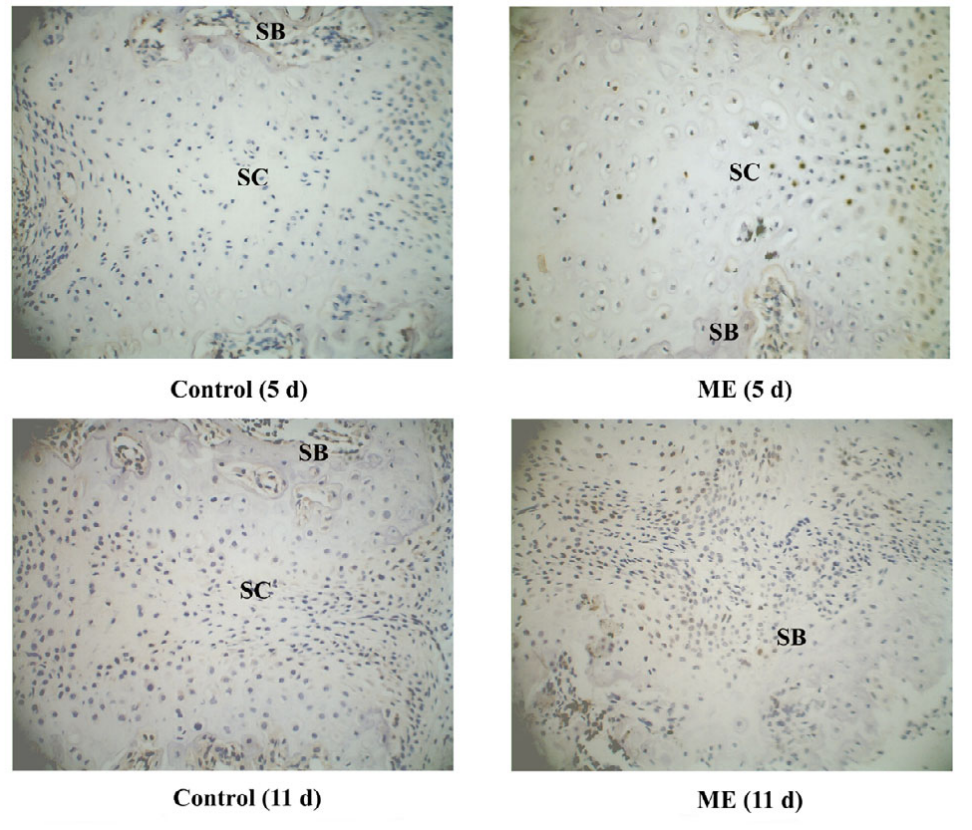
PCNA expression in the midpalatal suture. SC: suture cartilage. SB: suture bone. On day 5, some of the chondrocytes in the proliferating layer of the suture cartilage and osteoblasts at the end of the cartilage were PCNA-positive in the ME group. On day 11, many osteoblasts adjacent to the bone trabeculae were positive in the ME group. (Original magnification ×40).

**Figure 5:**
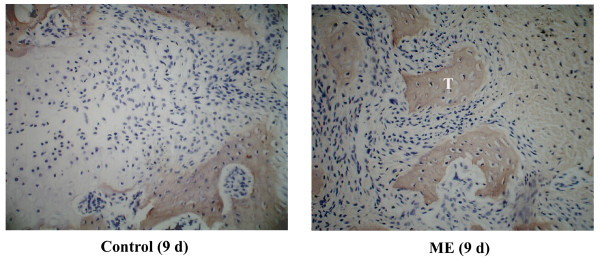
Collagen I expression in the expanded midpalatal suture 9 days after operation. The tissue in the suture (marked "T") from Figure 2H of the ME group is Collagen I-positive, indicating that it is bone, and not residual suture cartilage. (Original magnification ×40).

During the first three days, few PCNA-positive cells were observed in either group. From day 5, some of the chondrocytes in the inner layer of suture cartilage and osteoblasts at the end of the suture cartilage were PCNA positive in the ME group (Figure 4). On day 11, many osteoblasts adjacent to bone trabeculae were positive in the ME group (Figure 4). To identify the tissue (marked with "T") in the suture shown in Figure 2H of the ME group, we performed immunohistochemical analysis of Collagen I and found that it was bone tissue, but not residual suture cartilage (Figure 5).

### 2-DE, MALDI-TOF MS and protein identification

**Figure 6:**
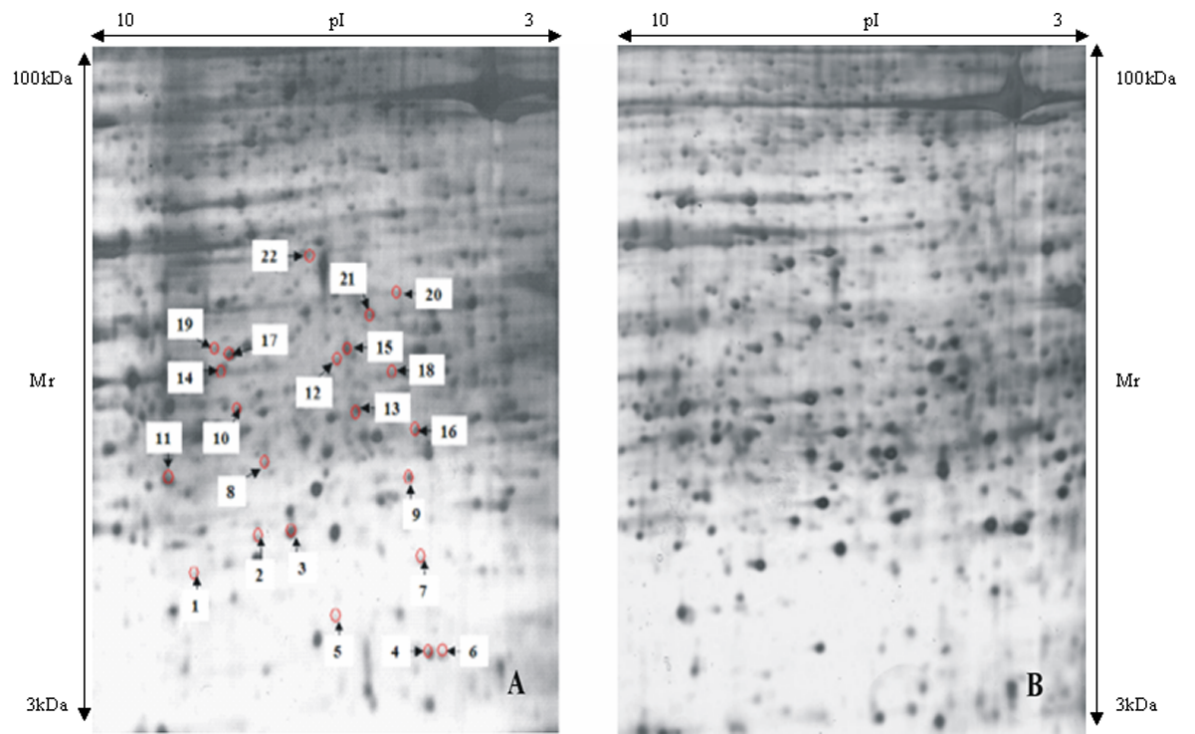
A: 2-DE map from an expanded midpalatal suture in the ME group. The labeled proteins are differentially expressed. B: 2-DE map from a midpalatal suture in the control group. (pI: Isoelectric point. kDa: kilodalton. Mr: Molecular weight).

There were 1862 (SD = 51.35) and 1944 (SD = 73.96) protein spots observed in gels from the control and ME groups, respectively. The number of spots detected in each set of three replicates was reproducible (coefficient of variation, CV 13–29%), as was their spot intensity (CV 21–39%). Following analysis with the PDQuest software, we selected 22 spots where the differentially-expressed proteins were more abundant in the ME group, for further analysis with MALDI-TOF MS. These 22 spots are marked with arrows in Figure 6. The proteins contained in the 22 spots were identified by MALDI-TOF MS on the basis of peptide mass matching. The identified proteins are listed in Table 1.

**Table 1:** Changes in protein expression observed in the expanded midpalatal suture.

Spot No.	Identified Protein	Accession Number	Calculated pI value	Nominal mass (Mr)	Number of Matched Peptides	Sequence Coverage	Fold Change	Mascot Score	Functional Category
2	Parathyroid hormone (PTH)	P04089	9.63	12722	7	71%	4.4	60	Osteogenesis
10	Annexin A2 (Annexin II) (Lipocortin II)	Q07936	7.53	38808	21	53%	10.2	131	Osteogenesis
11	Cartilage glycoprotein 39 (GP-39)	Q9WTV1	8.93	42708	5	28%	3.6	54	Osteogenesis
14	Osteoprotegerin (OPG)	O08727	8.79	47588	14	24%	4.1	57	Osteogenesis
21	Elongation factor 2 (EF-2)	P05197	6.42	96061	12	15%	42.8	59	Osteogenesis
6	GTP-binding protein RAB-3D	Q63942	4.75	24503	5	35%	3.0	57	Tissue resorption
18	Serine/threonine-protein kinase (PAK 1)	P35465	5.63	60825	10	27%	8.2	83	Tissue resorption and reconstruction of cytoskeleton
22	Deubiquitinating protein VCIP135	Q8CF97	6.77	135793	15	15%	3.6	55	Tissue resorption
5	Transgelin 2 (SM 22β)	Q5XFX0	8.41	22550	9	34%	14.3	54	Angiogenesis
7	Growth factor receptor-bound protein 2 (GRB2 adapter protein)	P62994	5.89	25304	7	40%	23.6	63	Angiogenesis
16	Vimentin	P31000	5.06	53626	13	27%	3.1	65	Angiogenesis and reconstruction of cytoskeleton
20	78 kDa glucose-regulated protein precursor (GRP 78)	P06761	5.07	72473	17	21%	3.8	100	Unfolded protein response
15	Adenylyl cyclase-associated protein 1 (CAP 1)	Q08163	7.30	51726	11	24%	4.0	54	Stress reaction and reconstruction of cytoskeleton
4	Adenylate kinase isoenzyme 1 (ATP-AMP transphosphorylase) (AK1)	P39069	7.71	21645	9	44%	3.7	62	Energy metabolism
8	L-xylulose reductase	Q920P0	6.82	25931	8	38%	6.5	61	Energy metabolism
12	Phosphoglycerate kinase 1	P16617	7.52	44794	10	29%	5.8	59	Energy metabolism
13	Alpha-enolase (ENO-1)	AAH63174	6.16	47309	13	34%	7.6	79	energy metabolism
17	ATP synthase alpha chain	P15999	9.22	59831	20	30%	4.8	110	Energy metabolism
1	Forkhead box protein D4 (FOXD4)	Q63249	10.61	11905	5	59%	5.2	52	Cell proliferation
3	PP5-TPR variant	Q80UF5	8.45	14325	8	56%	4.9	55	Cell proliferation
9	Psmd8 protein	AAI05895	6.05	32545	11	31%	4.3	66	Protein synthesis
19	APOBEC1 complementation factor	Q923K9	8.81	65864	10	20%	6.6	61	RNA editing

pI = Isoelectric point. Mr = Molecular weight

### Western blot analysis

**Figure 7:**
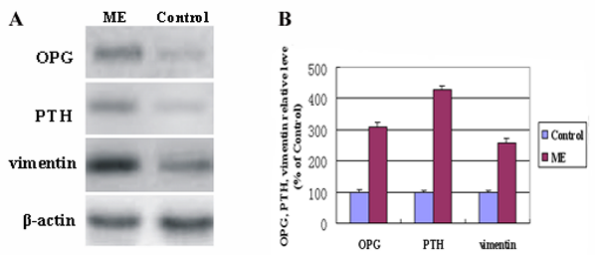
A: Representative Western blots for the expression analyses of OPG, PTH and vimentin. β-actin was used as the loading control. (OPG: osteoprotegerin. PTH: parathyroid hormone). B: The OPG, PTH and vimentin protein levels relative to β-actin protein levels were assessed by densitometric analysis, and expressed in the ME group relative to the same three proteins in the control group. (*The level in ME animals was significantly higher than the respective controls, *P *< 0.01).

As illustrated in Figure 7, the levels of PTH, OPG and vimentin proteins in the midpalatal suture tissues of the ME group were confirmed to be significantly higher than those in the control group (P < 0.01).

## Discussion

When stress is loaded onto the midpalatal suture, the mechanical force is transformed into a biological signal that results in a series of biological events, such as chondrocyte hypertrophy, angiogenesis, cartilage and other tissue resorption, skeletal matrix formation, and matrix calcification. Finally, mechano-transducted osteogenesis occurs and the maxillary arch is expanded. Numerous proteins are involved in these processes, and there is an urgent need to identify and characterize these critical proteins. Proteomics is the study of the function of all expressed proteins, i.e., the study of the proteome. Compared with the genome, the proteome not only varies by cell type, but also is dynamic in different physiological states within the same cell.

Rats are often used as an animal model of ME [2,4,6] based on economical and technical considerations. In the present study, there was an additional reason to use the rat model: contrary to the paucity of proteomic databases applicable to some other animals such as dogs and cats, the availability of the rat proteomic database would allow us to identify interesting proteins based on the MS results. The present study was undertaken to elucidate the changes in the proteome between non-stressed suture tissues and their counterparts subjected to mechanical stresses. The data compiled in Table 1 suggest that several biological processes were affected. The selected proteins that may participate in the up-regulation of these biological processes are discussed below.

### Osteogenesis

There are two main types of osteogenesis, endochondral and intramembranous ossifications. Some investigators suggest that intramembranous ossification is important to mid-palatal suture expansion [10]. Based on HE staining and immunohistochemical analysis of PCNA, we found that following exposure to mechanical stress, osteoblasts proliferated between the cartilage and periosteum. This cartilage was gradually absorbed and replaced by bone and connective tissue, indicating that intramembranous ossification by osteoblasts from the palatal periosteum plays an important role in ME. In addition, using Masson's staining, we observed considerable skeletal matrices adjacent to the suture cartilage, suggesting that endochondral ossification contributes to osteogenesis in the early stage of ME. Thus, both types of osteogenesis participate in the tissue remodeling process during ME.

Several proteins, which were observed to change following exposure to the mechanical stress, deserve additional discussion. First, osteoprotegerin (OPG) is a soluble 'decoy' receptor that binds to RANKL (Receptor Activator for NF-kB Ligand) and prevents it from binding to RANK (Receptor Activator for NF-kB) [11]. This effectively inhibits RANKL-mediated osteoclast maturation. The balance between the osteoclast-promoting RANKL and the osteoclast-inhibiting OPG can regulate the number and activity of osteoclasts. Annexin A2 (Annexin II, ANXA2) is a member of the calcium-dependent phospholipid-binding protein family. It regulates cell growth, and is involved in signal transduction pathways. It is also important for alkaline phosphatase activity in bone and is associated with osteoblast mineralization [12].

Parathyroid hormone (PTH) is produced almost exclusively by the parathyroid glands and is transported to target tissues through blood circulation. The anabolic action of PTH on cortical and cancellous bone has been validated [13]. The increased PTH levels in ME tissues may be associated with increased local vascular density and/or a rising concentration of serum PTH. Nevertheless, while PTH likely plays a role in tissue remodeling, it remains to be determined whether circulating endocrine hormones (such as PTH) or local cytokines (such as OPG and bone morphorgenic protein (BMP)) play the initial and key role in ME.

Elongation factor 2 (EF 2) belongs to the G-protein superfamily and catalyzes the translocation step of translation elongation after peptide bond formation occurs. It contributes to the differentiation of chondrocytes to the hypertrophic stage, which is essential for endochondral ossification [14]. Its presence suggests participation of endochondral ossification in osteogenesis during ME.

Cartilage glycoprotein 39 (GP-39) is produced by several types of human cells, including chondrocytes, synovial cells, macrophages, and neutrophils [15]. It is highly expressed in osteoarthritic synovial fluids and osteophytic tissues and may be a more accurate marker of chondrocyte activation in the disease process. This protein may participate in endochondral ossification and matrix resorption.

These five proteins were all up-regulated in the early stage of ME, and at least some of them may be responsible for the effects of ME. Future studies are needed to determine the effects of these on various types of osteogenesis, such as distraction osteogenesis in long bones, and the healing of bone fractures.

### Tissue resorption

In many circumstances, new bone formation is accompanied by bone resorption, and osteogenesis results from a change in the equilibrium between osteoclasts and osteoblasts. In the earlier stage of ME, resorption predominates, and some of the proteins listed in Table 1 may be important for this process. For instance, PAK1 can mediate osteoclasts through its involvement in the organization of the actin cytoskeleton, thereby regulating their resorptive activity [16]. In the present study, we found that PAK1 was up-regulated, suggesting increased osteoclast activity. Ras-related protein (RAB-3D) is involved in vesicular traffic and regulates the release of catabolic enzymes during bone resorption [17]. Deubiquitinating protein (VCIP135) is also involved in vesicular traffic [18]. The mechanisms by which the resorption of other tissues is regulated in the mid-palatal suture are not clear, and further studies of how these two proteins participate in tissue resorption are required.

### Angiogenesis

Angiogenesis is a pivotal process in osteogenesis, which regulates cell proliferation, differentiation, and bone formation and resorption. Transgelin 2 is one of the earliest markers of differentiated smooth muscle, and is expressed in smooth muscle cells and mesenchymal stem cells. Vimentin is a member of the intermediate filament family of proteins. Both transgelin 2 and vimentin have been identified as proteins that regulate angiogenesis [19-21]. Overexpression of GRB2, another molecule identified during our study, enhances VEGF-dependent cell migration and is regarded as a regulator of VEGF [22]. Based on these findings, we speculate that the induction of these three pro-angiogenic genes may support new vasculature for bone induction during ME.

### Other processes

#### Unfolded protein response (UPR) and stress reaction

UPR is a function of the endoplasmic reticulum (ER) that retains and finally degrades unfolded or mis-folded proteins, and provide strict quality control to ensure that only properly folded proteins are transported to the Golgi apparatus. UPR activity is regulated by a complex system, which includes the 78 kDa glucose-regulated protein GRP78 [23]. Cyclase-associated protein (CAP) is a multifunctional protein related to the stress reaction. The N-terminal region is required for RAS2Val-19-dependent heat shock sensitivity, while the C-terminal region is required for normal cell morphology and responsiveness to nutrient deprivation and excess [24]. Since ME is the application of mechanical stress to skeletal sutures followed by the induction of osteogenesis, an ER stress reaction should be one of the first processes induced by the mechanical stress. In the present study, the up-regulation of CAP that we observed may be an indication of the stress reaction, and the expression of GRP78 in the ME group indicates that this may be critical during the early period of expansion in order to ensure the proper assembly of proteins and, ultimately, the proper function of suture cells.

#### Energy metabolism

There are five proteins listed in Table 1 that are involved with energy metabolism including L-xylulose reductase [25], alpha-enolase (ENO1) [26], adenylate kinase isoenzyme 1 (AKI) [27], phosphoglycerate kinase 1 [28], and ATP synthase [29]. Many physiological processes are accompanied by an increase in energy metabolism, including tissue resorption, angiogenesis and osteogenesis, such as are observed during ME. Virtually all animal cells and tissues shift to some extent from mitochondrial respiration to anaerobic glycolysis for energy production when exposed to hypoxic conditions. The pattern of respiration/glycolysis during ME should be further examined to determine the effects of this stress on the suture cells.

#### Cell proliferation

Proliferation and migration of mesenchymal stem cells, osteoclasts, osteoblasts and fibroblasts are prerequisites for tissue resorption, angiogenesis and osteogenesis in ME. In the present study, on the fifth day after the operation, part of the chondrocytes in the proliferating layer of the suture cartilage, and the osteoblasts at the end of the cartilage were PCNA-positive in the group undergoing ME. On the eleventh day after the operation, many osteoblasts adjacent to the bone trabeculae were positive in the ME group. The proliferation of these cells was mainly related to osteogenesis. Protein serine/threonine phosphatase PP5 is the prototype of the fourth subfamily of the PPP family of protein phosphatases. Up-regulation of PP5 in rapidly growing cells, as compared to serum-deprived cells, suggests that it has a role in cell growth [30]. FOXD4 is a novel winged helix transcription factor belonging to the Forkhead-box (FOX) family, which plays an important role in embryonic development, cell cycle regulation, and oncogenesis [31]. These proteins might initiate or promote the proliferation of cells such as chondrocytes and osteoblasts.

#### Reconstruction of the cytoskeleton

As a key component of the intermediate filaments cytoskeleton, vimentin contributes to the maintenance of cell shape, migration, and response to mechanical stress [32]. p21-activated kinase 1 (PAK1) and cyclase-associated protein (CAP) also participate in the construction of the cytoskeleton [33,34]. In the mechanically-expanded mid-palatal suture, the cytoskeleton should function in the various processes of tissue remodeling, although the underlying mechanism is still unclear.

## Conclusion

Alterations in proteins and protein abundance, which encompass numerous cellular protein networks and functional networks, occur in the expanded midpalatal suture following ME. The profile of protein alterations suggests that the changes in the unfolded protein response and stress reaction, energy metabolism, angiogenesis, cell proliferation, reconstruction of the cytoskeleton, tissue resorption, and osteogenesis are involved in tissue remodeling during maxillary expansion. Moreover, many of these proteins have not been reported in skeletal tissues, and understanding their functions requires further study in order to determine their potential for developing optimal clinical treatment approaches.

## Competing interests

The author(s) declare that they have no competing interests.

## Authors' contributions

LW and JM conceived of the study, participated in the design of the study and performed the statistical analyses. All authors carried out the experiments. JM drafted the manuscript with the help of RJS and LW. All authors have read and approved the final manuscript.

## Pre-publication history

The pre-publication history for this paper can be accessed here:


